# Practical Observations on the More Obstinate and Inveterate Venereal Complaints

**Published:** 1785

**Authors:** 


IV. *Practical obſervations on the more obſtinate and inveterate venereal complaints. By* F. Swediar, *M. D.* 8vo. Johnſon, *London*, 1784. 233 pages.

NO branch of the Medical art has, perhaps, of late years, received ſo many valuable valuable improvements as the treatment of the venereal diſeaſe ; and to theſe improvements it is probably owing that the effects of this malady, are, in general, leſs violent and fatal than formerly. The author of the work now before us is of opinion, that this diſeaſe is not only leſs frequent, but in all its different ſymptoms leſs violent, in this capital, than in any other in Europe. The reaſon of this, he thinks, is principally owing to the eaſe with which perſons of the lower claſs, labouring under this complaint, procure relief at hoſpitals or diſpenſaries. " Our Phyſicians and " Surgeons," he obſerves, " do not think " themſelves authoriſed to reproach their vene- " real patients with their miſery in a rude and " inhuman manner, or to let theſe poor crea- " tures ſuffer, in order to pleaſe God Almighty, " as inſtruments of his vengeance ; or think " themſelves appointed by Heaven to puniſh " rather than relieve them, as I have a thouſand " times heard even in great capitals in different " parts of the continent. Our magiſtrates and " police do not force theſe wretches into a " priſon, or into an hoſpital, not much diffe- " rent from a priſon, but put in their way all " poſſible means to procure relief for them-

 " ſelves.

" felves. In other countries, where government " purfues a different plan, where patients have " no place of refort, where they are expofed to " die of hunger during the cure, or where they " are even intimidated from applying in time, " in thofe countries I have frequently feen the " diforder in its moft horrid ftages, almoft un- " known in this country. In fhort, let a perfon " make the tour of Europe, and only take " notice of the venereal patients, as well thofe " who are confined in hofpitals, as thofe who " live or die unnoticed, under the moft horrid " fymptoms of this difeafe, in their private " abodes; and he will be able to form as folid " a judgement of the comparative progrefs of " enlightened principles of government in dif- " ferent countries, from thefe obfervations, as " from any other inquiry whatfoever."

After fome obfervations on venereal infection, and the different appearances of fyphilitic complaints in general, Dr. Swediar proceeds to treat of the gonorrhœa. He diffents from thofe writers who maintain that gonorrhœa and fyphilis are the effects of two diftinct poifons; and in reply to an affertion, that the inhabitants of the South-Sea iflands, though afflicted with the lues, are as yet free from the gonorrhœa, he brings brings the teſtimony of Captain King, who has aſſured him, that he himſelf has ſeen many of them with the matter dripping from their urethra.

In caſes of ſwelled teſticle, among other remedies, Dr. Swediar recommends warm bathing, clyſters, and opiates, as affording the moſt certain and ſpeedy relief.—He diſtinguiſhes venereal ulcers into local and univerſal. In the former of theſe he is averſe to the internal uſe of mercury. When a bubo has begun to form, he thinks we ſhould always attempt to diſcuſs it as ſoon as poſſible, provided no ſigns of ſuppuration have already made their appearance. In general, he leaves the opening of abſceſſes of this ſort to nature; but if a cauſtic is thought to be neceſſary, recommends the application of a very ſmall one, according to Mr. Plenck's method.

In the confirmed lues, when no particular circumſtances forbid it, he ſeems to prefer the method of cure by frictions, and the occaſional uſe of the warm bath. He thinks ſalivation not only unneceſſary but hurtful.

After treating of the venereal diſeaſe in general, the author proceeds to deſcribe ſome particular venereal complaints, as ophthalmia,

ſore

ſore throat, cutaneous eruptions, &c. which require a particular method of cure. He alſo treats of the complication of the venereal virus with other diſeaſes; and of caſes, for the cure of which mercury is inſufficient. Under each of theſe heads we meet with a variety of judicious obſervations.

